# Investigating silent pauses in connected speech: integrating linguistic, neuropsychological, and neuroanatomical perspectives across narrative tasks in post-stroke aphasia

**DOI:** 10.3389/fneur.2024.1347514

**Published:** 2024-04-12

**Authors:** G. Angelopoulou, D. Kasselimis, M. Varkanitsa, D. Tsolakopoulos, G. Papageorgiou, G. Velonakis, E. Meier, E. Karavassilis, V. Pantoleon, N. Laskaris, N. Kelekis, A. Tountopoulou, S. Vassilopoulou, D. Goutsos, S. Kiran, C. Weiller, M. Rijntjes, C. Potagas

**Affiliations:** ^1^Neuropsychology&Language Disorders Unit, 1st Department of Neurology, Eginition Hospital, Faculty of Medicine, National and Kapodistrian University of Athens, Athens, Greece; ^2^Department of Psychology, Panteion University of Social and Political Sciences, Athens, Greece; ^3^Center for Brain Recovery, Boston University, Boston, MA, United States; ^4^2nd Department of Radiology, General University Hospital “Attikon”, Medical School, National and Kapodistrian University of Athens, Athens, Greece; ^5^The Aphasia Network Lab, Department of Communication Sciences and Disorders, Northeastern University, Boston, MA, United States; ^6^School of Medicine, Democritus University of Thrace, Alexandroupolis, Greece; ^7^Department of Industrial Design and Production Engineering, School of Engineering, University of West Attica, Athens, Greece; ^8^Stroke Unit, 1st Department of Neurology, Eginition Hospital, National and Kapodistrian University of Athens, Athens, Greece; ^9^Department of Linguistics, School of Philosophy, National and Kapodistrian University of Athens, Athens, Greece; ^10^Department of Neurology and Clinical Neuroscience, University Hospital Freiburg, Freiburg, Germany

**Keywords:** post-stroke aphasia, silent pauses, stroke story, picture description, neuropsychological performance, dorsal stream, ventral stream

## Abstract

**Introduction:**

Silent pauses are regarded as integral components of the temporal organization of speech. However, it has also been hypothesized that they serve as markers for internal cognitive processes, including word access, monitoring, planning, and memory functions. Although existing evidence across various pathological populations underscores the importance of investigating silent pauses’ characteristics, particularly in terms of frequency and duration, there is a scarcity of data within the domain of post-stroke aphasia.

**Methods:**

The primary objective of the present study is to scrutinize the frequency and duration of silent pauses in two distinct narrative tasks within a cohort of 32 patients with chronic post-stroke aphasia, in comparison with a control group of healthy speakers. Subsequently, we investigate potential correlation patterns between silent pause measures, i.e., frequency and duration, across the two narrative tasks within the patient group, their performance in neuropsychological assessments, and lesion data.

**Results:**

Our findings showed that patients exhibited a higher frequency of longer-duration pauses in both narrative tasks compared to healthy speakers. Furthermore, within-group comparisons revealed that patients tended to pause more frequently and for longer durations in the picture description task, while healthy participants exhibited the opposite trend. With regard to our second research question, a marginally significant interaction emerged between performance in semantic verbal fluency and the narrative task, in relation to the location of silent pauses—whether between or within clauses—predicting the duration of silent pauses in the patient group. However, no significant results were observed for the frequency of silent pauses. Lastly, our study identified that the duration of silent pauses could be predicted by distinct Regions of Interest (ROIs) in spared tissue within the left hemisphere, as a function of the narrative task.

**Discussion:**

Overall, this study follows an integrative approach of linguistic, neuropsychological and neuroanatomical data to define silent pauses in connected speech, and illustrates interrelations between cognitive components, temporal aspects of speech, and anatomical indices, while it further highlights the importance of studying connected speech indices using different narrative tasks.

## 1 Introduction

Connected speech may serve as a valuable resource in language research, offering a wide range of quantitative and qualitative metrics derived from diverse speech genres; their examination has the potential to provide significant insights into the language and other cognitive abilities involved in speech output. Notably, narrative tasks (i.e., any elicitation task, which can be used to obtain a speech sample from an individual, including -but not limited to- free narration, storytelling, picture description, etc.) enhance the ecological validity of language assessment by closely simulating the natural language behavior observed in individuals’ daily lives, as opposed to the standardized neuropsychological tasks (1). Growing evidence put forward the notion that the temporal characteristics of speech, particularly the frequency and duration of silent pauses (i.e., the empty/silent gaps interrupting speech), may serve as a direct mean for the online investigation of the underlying cognitive processes that support speech production [see for example: (3) for post-stroke aphasia; (4) for Primary Progressive Aphasia (PPA); (5) for Alzheimer’s Disease; (6), for patients with schizophrenia]. Such processes include access to semantic representations, retrieval of the accurate target word, monitoring, selective retrieval of information from episodic/semantic memory, and the planning and organization of speech output, while the cognitive effort may vary depending on the demands of distinct speech genres.

A thorough literature search uncovers limited studies directly investigating the occurrence of silent pauses in patients with aphasia. Since the inaugural studies (7–9), contemporary research has revisited the importance of exploring silent pauses, with a particular focus on specific aspects such as frequency and duration (10) within distinct aphasia profiles (11). However, in these studies, very small sample sizes were assessed in district narrative tasks, highlighting the need for more extensive and inclusive investigations in this domain.

In a recent study conducted by our team (3), we explored the duration patterns of silent pauses in a free narrative task, meaning a personal story, with no visual stimuli restrictions, involving 18 patients with chronic post-stroke aphasia. Our data supported the hypothesis of a binomial distribution of silent pause duration in both populations, aligning with the proposition by Kirsner et al. (10). This implies that silent pauses’ duration comprises two distributions, one characterized by shorter durations and another by longer durations. Moreover, we proposed a potential association between longer pauses and word-finding deficits. DeDe and Salis (12) contributed to the understanding of speech fluency in patients with aphasia, using a different narrative task, i.e., a fairy tale. Patients with anomic aphasia and patients with latent aphasia, that is, patients with subtle or minimal language impairments (13), exhibited significantly longer pauses compared to healthy speakers. Interestingly, the healthy speakers’ group produced significantly shorter pauses when introducing a new episode, when compared with the two clinical groups, meaning patients with anomic and latent aphasia. The authors assumed that this discrepancy may indicate a disparity in processing speed during the planning of a new oral sentence. Harmon et al. (14) have illustrated that ratio of extended and filled pauses per utterance, in a story retelling task, can effectively differentiate patients with moderate aphasia from those with severe aphasia. Lastly, Feenaughty et al. (15) reported that patients with non-fluent post-stroke aphasia conducted longer pauses compared to patients with fluent aphasia, yet no differences were reported for frequency.

It should be noted that in each of these studies, different speech genres were used, such as a personal story narration of stroke (2); a well-known (fairy tale) story narration (12) or a story retelling task (14). Interestingly, only Feenaughty et al. (15) compared the frequency and duration of silent pauses in a picture description and a speech entrainment task in a group of patients with fluent and non-fluent aphasia. A widely recognized yet insufficiently explored concept posits that different narrative tasks impose distinct linguistic demands, representing discrete cognitive tasks with varying levels of difficulty (16–18). For example, one would expect that a picture description task would require the engagement of cognitive mechanisms similar to those that are essential for confrontational naming, whereas a personal story narration would involve additional cognitive domains, including episodic memory. Consequently, various indices obtained through connected speech quantification may be distinguished based on the implemented elicitation technique. Despite limited evidence regarding post-stroke aphasia patients’ performance in elicitation tasks involving different speech genres in other indices [see, for example, (18, 19)], to the best of our knowledge, no prior study has directly investigated the frequency and duration of silent pauses in diverse narrative tasks.

Finally, there is currently insufficient evidence demonstrating direct correlations between silent pause characteristics and performance in neuropsychological tests among patients with post-stroke aphasia. There have been some indications suggesting relationships between silent pauses’ frequency in various narrative tasks (such as fairy tale narrations and pictures description tasks) and performance in word-finding tasks, such as naming tests and verbal fluency tests; however, these results are primarily drawn from evidence in patients with (PPA) (20) and patients with Alzheimer’s Disease (21). These findings in clinical populations with acquired language and other cognitive deficits emphasize the necessity to expand the investigation to patients with post-stroke aphasia. Likewise, the neural correlates of pauses’ measures in connected speech, have been sparsely investigated in patients with neurodigenerative disorders (5, 20, 21), yet no evidence so far exists with regard to patients with post-stroke aphasia.

Building upon our prior work on silent pauses in narrative speech of patients with aphasia (3, 4), the present study adopts an integrative approach to analyze silent pauses measures (i.e., frequency and duration) in two distinct narrative tasks and their correlation patterns with neuropsychological and neuroanatomical data. Based on initial theoretical models and studies suggesting that silent pauses’ function differentiates according to their duration, frequency and location in speech flow (22, 23) along with sparse evidence [see for example: (3, 12)], our hypothesis is that silent pauses reflect the internal cognitive processing of speech planning and word finding while speaking. Thus, we have selected two entirely different narrative tasks—a personal story (stroke story) and a picture description based on visual stimuli—assumed to rely on distinct cognitive processes, to investigate silent pauses’ measures.

First, we are going to investigate whether measures of silent pauses, meaning frequency and duration, differ in the two narrative tasks as a function of group (i.e., patients with aphasia versus healthy speakers). Based on our previous findings (3), we hypothesize that our patients will exhibit more silent pauses of larger duration compared to our healthy participants, while they will also indicate narrative task-related differences, as we have previously identified in individuals with PPA (4).

Second, we will investigate the potential cognitive role of silent pauses in aphasia. For this reason, we will focus on the patients’ data and incorporate silent pauses’ location (between-within clauses) in the specific models, investigating possible relations between silent pauses and performance in neuropsychological tasks. For this specific aim, our rationale is based on previous studies suggesting that pause location may reflect distinct cognitive processes (5, 24, 25, 26). Drawing from limited prior findings in patients with Alzheimer’s Disease (21), we anticipate that in the patients’ group, the association patterns between silent pauses’ measures and cognitive scores, will differentiate as a function of the narrative task.

Finally, we will explore whether characteristics of silent pauses in patients with aphasia can be predicted by lesions in specific language related regions of interest (ROIs) within the left hemisphere. Expanding upon our prior research findings on speech rate (19), we posit that silent pauses’ measures will show distinct association patterns with specific lesion loci, depending on the narrative task.

## 2 Materials and methods

### 2.1 Participants

Thirty-two patients (17 males) with post-stroke chronic aphasia (at least 6 months poststroke) following a single left hemisphere stroke, 25–75 years old (mean: 60.07, SD: 13.0) and 6–20 years of formal schooling (mean: 13.9, SD: 3.6) were recruited from the project “*Investigation of common anatomical substrate of linguistic and non-linguistic cognitive deficits in poststroke aphasia*,” conducted in the Neuropsychology and Language Disorders Unit of the First Neurology Department, Eginition Hospital, School of Medicine, National and Kapodistrian University of Athens (research protocol approval ID: ΩΣ3Ξ46Ψ8Ν2-00Φ, July 2017). Based on previously reported methodology (3), patients with global aphasia or increased dysfluency, as reflected in a speech rate index (words/min) lower than 30, were excluded, as severe speech production deficits would seriously hamper the purpose of the current study. All patients had a single left hemisphere stroke and were characterized as aphasic according to the Boston Diagnostic Aphasia Examination standard assessment (BDAE-SF) (27), adapted in Greek (28) (29, 30). Patients with further neurological and/or psychiatric disorders were excluded, according to recruitment guidelines of the project. Participants had no signs of dysarthria, based on clinical assessment conducted by an experienced speech and language pathologist (GP).

Sixty four healthy individuals (33 males), 25–65 years old (mean: 44.38, SD: 11.9) and 9–22 years of formal schooling (mean: 15.40, SD: 3.2) were recruited to formulate the control group, as part of the project “Investigation of cortical surface patterns and their relation with speech metrics and performance in neuropsychological assessment in healthy participants” also conducted in the Neuropsychology and Language Disorders Unit of the First Neurology Department, School of Medicine, National and Kapodistrian University of Athens, Eginition Hospital (research protocol approval ID: ΩOΞΛ46Ψ8N2 − 7PN, July 2017). Participants had no history of neurological and/or psychiatric disorders.

All participants were right-handed, monolinguals, native speakers of Greek. Informed consent was obtained from all participants prior to participation, according to the Eginition Hospital Ethics Committee (For detailed demographics, see Table 1).

**Table 1:** Demographic characteristics of the group of patients with aphasia and healthy participants.

	Patients with aphasia (*n* = 32)		Healthy Participants (*n* = 64)		*p*
	Range	Mean (SD)	Range	Mean (SD)	
Age (years)	25–75	60.07 (13.0)	25–65	44.38 (11.9)	<0.001^1^
Education (years)	6–20	13.90 (3.6)	9–22	15.40 (3.2)	ns
Sex		17 males		33 males	ns^2^
Time Post onset (months)	6–200	37.72 (50.9)			
Lesion Volume	5.97–177.4	57.25 (41.1)			

1 Independent samples t-test.

2 Chi-square.

### 2.2 Narrative ability assessment and connected speech analysis

Stroke story narration and “cookie theft” picture description were acquired for each participant during individual examination, as part of the Boston Diagnostic Aphasia Examination standard assessment (BDAE-SF; Goodglass and Kaplan, 1972), adapted in Greek (28). Healthy individuals were asked to describe a medical event of their own or of someone related to them, as a recount of a past event. Here we should acknowledge that the two elicitation tasks are not identical, since the control participants had not experienced a stroke. However, we argue that the description of a medical event by the healthy individuals is the closest equivalent to the stroke story; it should be also mentioned that it has been used as such in previous studies [see (3, 31)]. No time restrictions were applied, and participants were free to speak for as long as they wanted in both tasks. In the case of exceedingly brief narrations and/or the occurrence of long silences, the examiner provided minimum encouragement, through a standard set of questions, based on specific instructions.

Audio files were first undergone preprocessing with Audacity, version 2.4.2., to reduce audio noise and remove non-narrative parts (as examiner’s question and/or guidance). Silent pauses were annotated following the pipeline presented in Angelopoulou et al. (3), using ELAN program (Brugman and Russell, 2004; Wittenburg et al., 2006). Speech samples were orthographically transcribed following the basic conventions of the coding conventions of the Greek Aphasia Error Corpus [see (32)]. Silent pauses’ frequency was calculated for each participant, for each narrative task, adjusted for total number of words utterned. Individual values of silent pauses’ duration were extracted for each participant in milliseconds (ms), for both narrative tasks and were then transformed in log values, using the natural algorithm ln [see (3)]. Clause-like units were then identified by two rates. Clause like Units (CLU) were defined as a syntactically and/or prosodically marked stretch of speech containing one verb. It should be noted that due to the increased confounds in literature with respect to utterances’ annotation [see (33), for an extended discussion], it was decided to annotate speech on the basis of the simplest definition of clause, as presented above, following Goldman-Eisler (22) and Grande et al. (34), and not using utterances’ definition. The main aim was to annotate whether silent pauses occurred between or within clauses (see (35) for a detailed description of methodological pipeline of connected speech analysis).

### 2.3 Neuropsychological assessment

Neuropsychological testing of patients’ group consisted of the Boston Diagnostic Aphasia Examination standard assessment (BDAE-SF) (27), adapted in Greek (28), a confrontation picture naming test, the Boston Naming Test (BNT) (36), a receptive vocabulary test, the Peabody Picture Vocabulary Test – Revised (37), both adapted in Greek (38, 39), a test of complex commands, the Comprehension of Instructions in Greek (CIG) (40) and the Controlled Oral Word Fluency for Greek language (COWF) (41).

### 2.4 Neuroimaging data acquisition and analysis

Patients underwent a magnetic resonance imaging protocol on a 3 T Philips Achieva-Tx MR scanner (Philips, Best, Netherlands), equipped with an eight-channel head coil, at 2nd Department of Radiology of General University Hospital “Attikon,” located at Eginition Hospital in Athens.

The imaging acquisition protocols included:

T1-weighted sequence, repetition time = 9.90 ms; echo time = 3.69 ms; flip angle = 70o; 170 contiguous 1 mm slices; field of view = 250 × 250 mm; matrix size = 256 × 256, voxel size = 1 × 1 × 1 mm3; slice thickness = 1 mm. Moreover, FLAIR images were also included in the acquisition protocol.

T1 and Flair images were used in order to draw lesions in the left hemisphere. Lesion drawing was manually conducted in T1, projecting to Flair images in order to verify the lesion locus, using MRIcron^3^ (42), by two experienced neuropsychologists (DK and GA). All lesion maps were then visually inspected by an experienced neuroradiologist (GV) and an experienced neurologist (CP) blind to the patients’ language profile. T1 and lesion maps’ normalization was conducted as described in (43).

**Figure 1:**
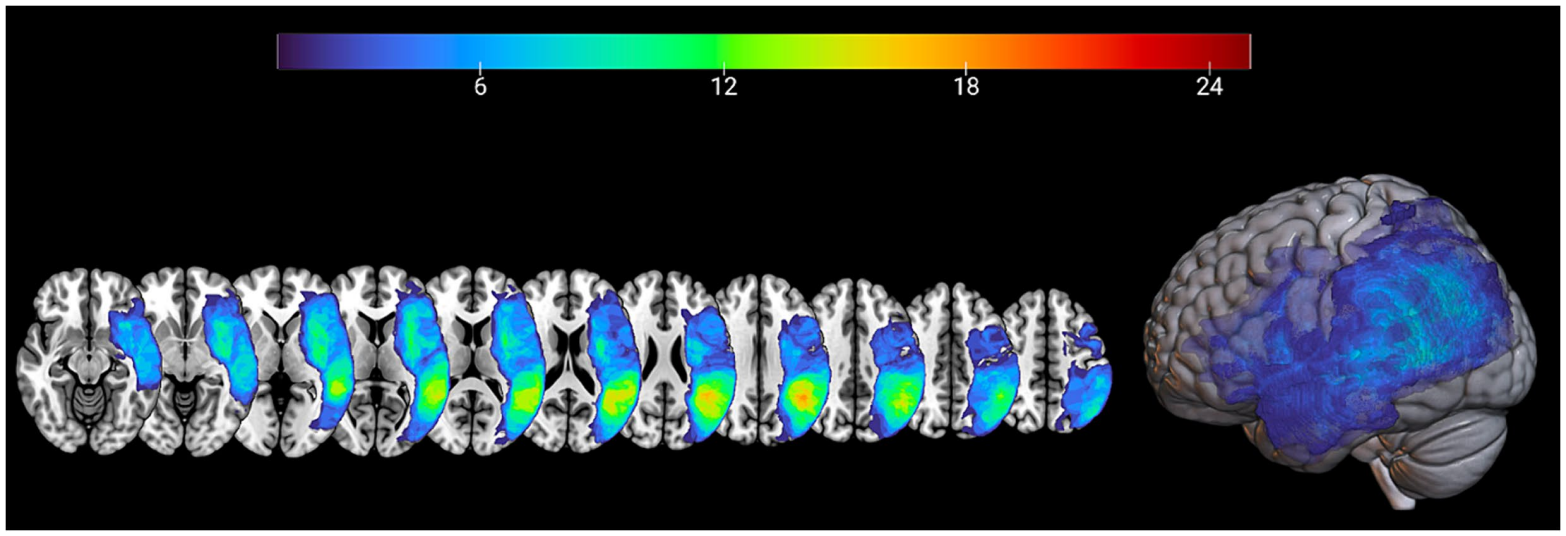
Lesion maps overlay for the patients’ group (*n* = 32) are shown on axial (left) and 3D view (right) of the standard average SPM152 brain template.

In order to calculate the percentage of spared tissue in specific regions of interest (ROIs) of the left hemisphere, the Automated anatomical labeling digital atlas was used (44). Regions of interest traditionally related with language processing [see for example: (45)] included: pars opercularis and pars triangularis of the Inferior Frontal Gyrus, Superior and Middle Temporal Gyrus, Insula and Inferior Parietal Lobe. All ROIs were thresholded with a probability value of 0.20, binarized, resampled and registered to the resolution and dimensions of patients’ normalized lesion maps. Spared tissue voxels for each ROI mask along with total lesion volume were calculated by using inhouse Matlab scripts [for a detailed description see (46, 47)] for further statistical analysis. The lesion overlap of the patients’ group is presented in MRIcronGL^4^ (see Figure 1 for lesion overlap).

### 2.5 Statistical analysis

All data were analyzed using the open-source statistical package R version 4.3.1 (R Development Core Team, 2011).^5^ Linear mixed-effects models were conducted using the “lmer” function [R package lme4; (48, 49)], implementing the Satterthwaite approximations for degrees of freedom (50, 51). Participants were used as random factors. The best-fit model was calculated each time, by identifying the model with the lowest Akaike Information Criterion (AIC).

Marginal and Conditional r square of each model were calculated via the r.squaredGLMM function of the “MuMIn” package (52, 53). Alpha level of 0.05 was used for all statistical tests.

All analyses were conducted using silent pauses’ frequency and duration. More specifically, for our first research question we conducted linear mixed effects models, using the lme4 package [R package lme4; (48, 49)], to compare silent pauses’ characteristics (frequency and duration) derived from healthy participants and patients with aphasia, in the two narrative tasks. Task (stroke story vs. picture description) and group (patients with aphasia vs. healthy adults) were included in the models as the fixed factors. An interaction between these two factors was also included. In total, we run two models, one for silent pauses duration and one for frequency. When significant, interactions were followed up with the “emmeans” package. Benjamini and Hochberg’s (54) false discovery rate (*p* < 0.05) were applied to correct for multiple comparisons.

For our second aim, only patient data were used. For silent pauses’ duration, linear mixed effects models, using the lme4 package [R package lme4; (48, 49)], including every single pause’s duration as a dependent variable, were conducted. Performance on the neuropsychological assessments (i.e., the BNT, the receptive vocabulary task, the semantic and phonemic fluency and the CIG), task (stroke story vs. picture description), and location of silent pauses (between or within clauses) were included in the models as fixed factors. We also looked at the interaction between performance on the neuropsychological assessments and task as well as between performance on the neuropsychological assessments and location. In total, five distinct models were run, one for each neuropsychological assessment. The best fitting model included demographic variables, such as age, years of formal schooling, sex, along with total duration of narration as covariates. A similar set of five statistical models was conducted, with fixed and random factors entered as described above and frequency of silent pauses serving as a dependent variable.

For our third aim, for silent pauses’ duration, linear mixed effects models, similar to those used for the second aim were conducted. In the first model, regions which correspond to the dorsal stream of language (45), that is pars opercularis of the Inferior Frontal Gyrus, Superior Temporal Gyrus and Inferior Parietal Lobe, were included as fixed factors, while in the second model regions which correspond to the ventral stream of language (45), that is pars triangularis of the Inferior Frontal Gyrus, Middle Temporal Gyrus and Insula were included as factors. Interaction of the Regions of Interest with narrative task was entered in each model to identify whether the effect of Regions of Interest spared tissue on silent pauses depends on the narrative task. The best fitting model included demographic variables, such as age, sex and total lesion volume as covariates. A similar set of two statistical models was conducted, with fixed and random factors entered as described above and frequency of silent pauses serving as a dependent variable.

## 3 Results

### 3.1 First research question

**Figure 2:**
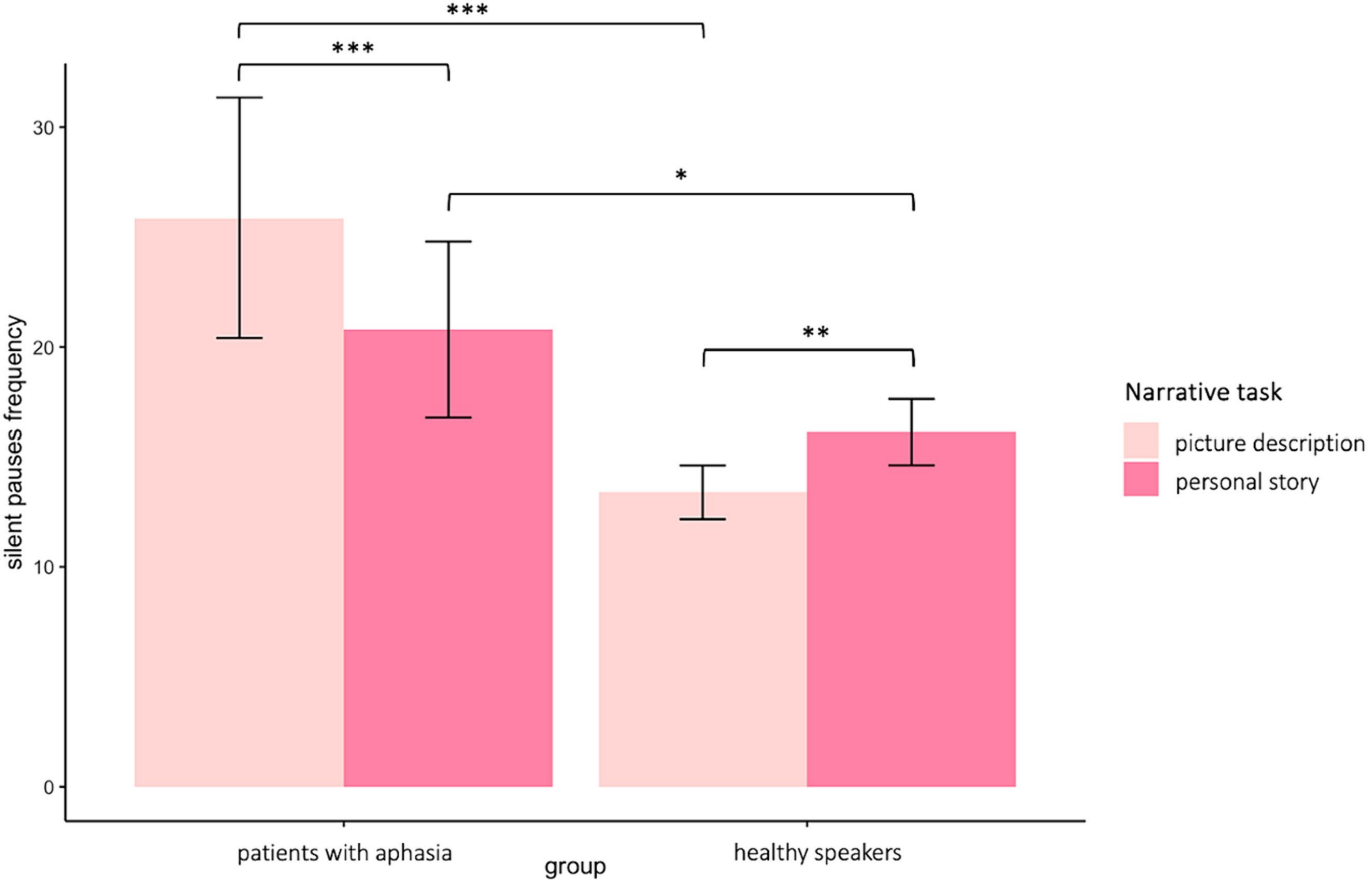
Error bar charts indicating silent pauses’ duration in patients with aphasia and healthy participants in the two narrative tasks, picture description and personal story. *p < 0.05, **p < 0.01, ***p < 0.001.

A significant interaction appeared between narrative tasks and participants’ groups in the model for silent pauses’ frequency. Patients with aphasia exhibited significantly increased pause frequency as compared to healthy controls in both picture description and personal story. Within group comparisons revealed that patients produced significantly more pauses in picture description, as compared to personal narration. Healthy controls indicated significantly increased pause rate in personal story, as compared to picture description (see Table 2 for speech and silent pauses’ measures and Table 3—model (1) for the pause frequency model; Figure 2 for error bar chart).

**Table 2:** Descriptive statistics for linguistic elements for the groups of patients with aphasia and healthy participants.

	Patients with aphasia (*n* = 32)		Healthy Participants (*n* = 64)	
	Range	Mean (SD)	Range	Mean (SD)
Picture description				
Total number of words	15–103	65.55 (25.5)	33–108	71.05 (24.7)
Total number of silent pauses	4–26	14.72 (6.1)	2–16	9.25 (3.5)
Silent pauses’ frequency^1^	6.80–71.88	25.87 (14.4)	4.85–26.42	13.41 (4.9)
Silent pauses’ duration (ms)^2^	445.14–3640.61	1293.37 (740.0)	428.93–2515.33	927.07 (360.8)
Silent pauses’ duration (log)^3^	6.10–8.20	7.03 (0.5)	6.06–7.83	6.77 (0.3)
Personal story narration				
Total number of words	31–110	89.59 (23.8)	31–111	95.11 (15.9)
Total number of silent pauses	6–37	17.21 (7.4)	4–30	14.68 (5.4)
Silent pauses’ frequency^1^	6.12–56.86	20.79 (10.6)	3.96–32.26	16.13 (6.0)
Silent pauses’ duration (ms)^2^	333.50–2208.32	1008.09 (458.3)	537.36–1602.20	931.06 (234.1)
Silent pauses’ duration (log)^3^	5.81–7.70	6.83 (0.40)	6.29–7.38	6.81 (0.2)

1 Adjusted for total number of words.

2 Values in milliseconds.

3 Values transformed using natural logarithm ln.

**Table 3:** Linear mixed effects models for silent pauses frequency and duration in healthy participants and patients with aphasia.

Model for silent pauses frequency					
	Fixed effects				
	Est	SE	95%CI	t	*p* value
Intercept	25.877	1.6	12.07–14.73	**16.698**	<0.001
Narrative task	−5.084	1.2	1.27–4.00	**−4.274**	<0.001
Group	−12.470	1.9		**−6.675**	<0.001
Narrative task x Group	7.808	1.4		**5.445**	<0.001
	Random effects				
	Variance		SD		
Participant (intercept)	49.12		7.01		
	Model fit				
	Marginal		Conditional		
R^2^	0.22		0.77		
Model for silent pauses duration					
Intercept	6.610	0.04	12.07–14.73	**173.726**	<0.001
Narrative task	0.066	0.03	1.27–4.00	**2.050**	0.040470
Group	0.219	0.06		**3.407**	0.000848
Narrative task x Group	−0.221	0.05		**−4.275**	<0.001
Participant (intercept)	0.05		0.23		
R^2^	0.22		0.77		

**Figure 3:**
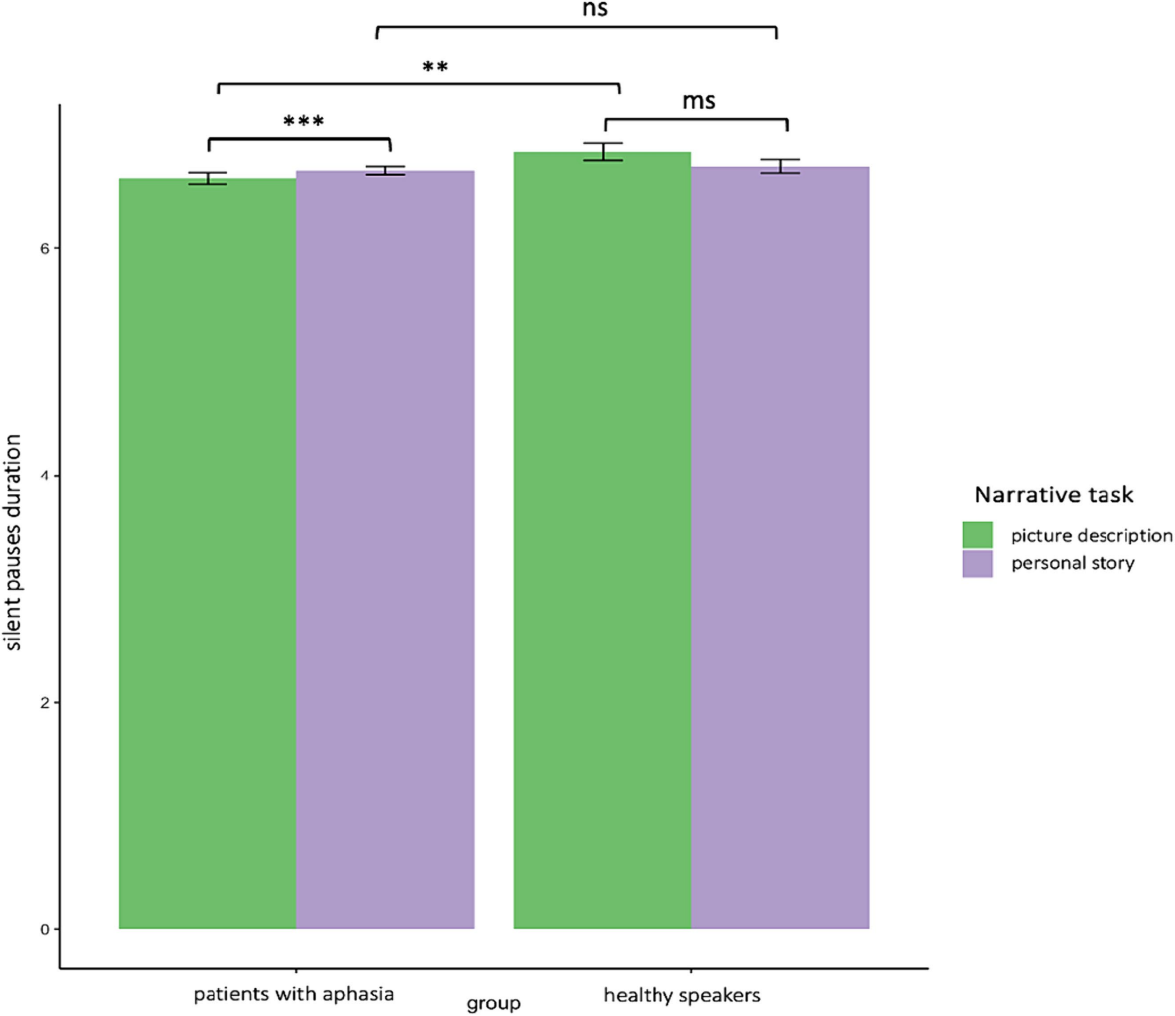
Error bar charts indicating silent pauses’ duration in patients with aphasia and healthy participants in the two narrative tasks, picture description and personal story. *p < 0.05, **p < 0.01, ***p < 0.001.

Similarly, a significant interaction appeared between narrative tasks and participants’ groups in the second model for silent pauses’ duration. Patients with aphasia produced significantly longer pauses, as compared to healthy controls only in picture description. In the personal story, patients indicated longer pauses compared to healthy speakers, yet this difference was not significant. Within group comparisons revealed that patients produced significantly longer pauses in picture description, as compared to personal narration. Healthy controls produced longer pauses in personal story, compared to picture description, yet this difference was marginally significant (see Table 3—model (2) for the pause duration model; Figure 3 for error bar chart).

### 3.2 Second research question

**Figure 4:**
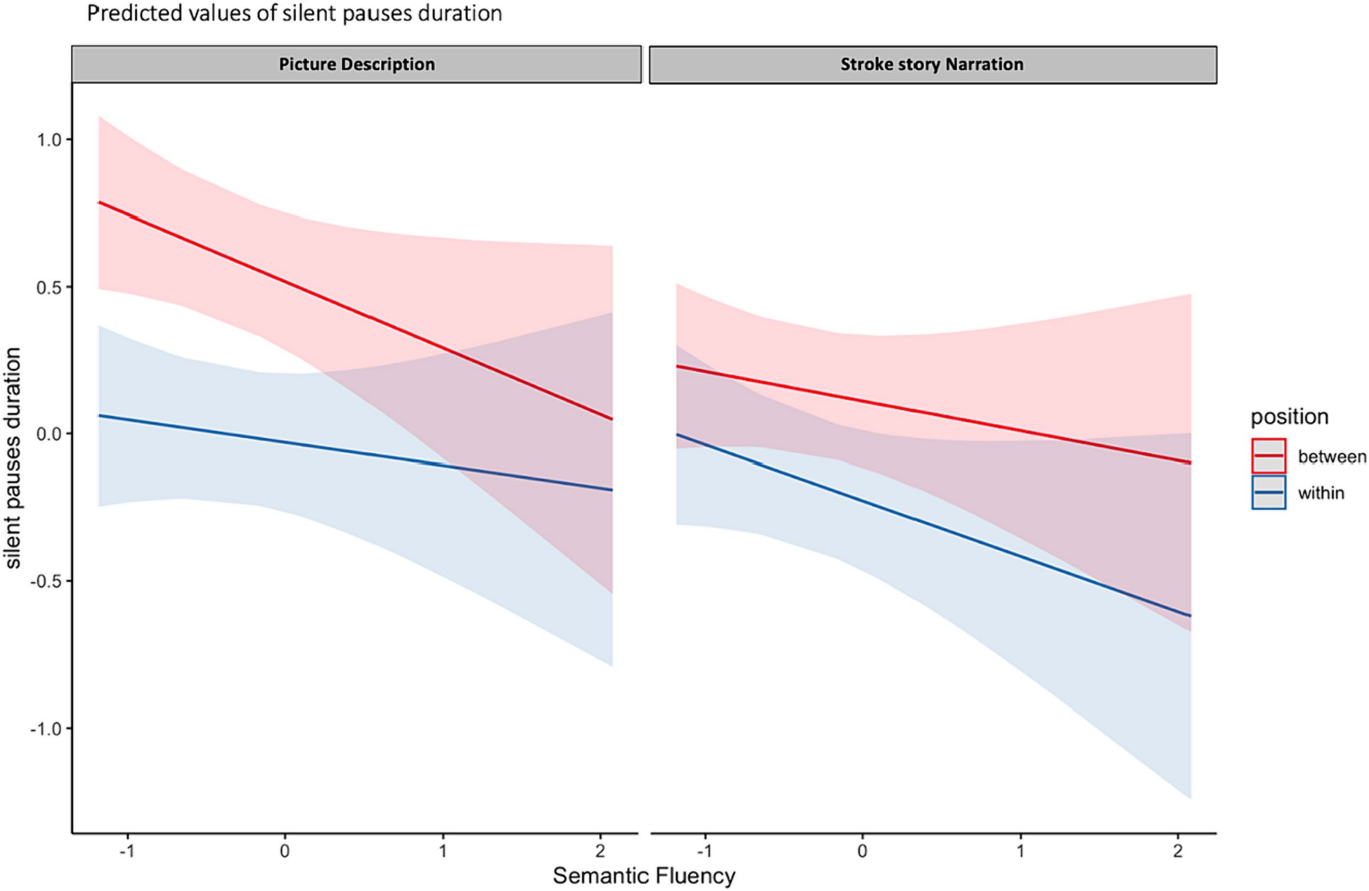
Effects plot for three-way interaction among narrative task, performance in SVF and silent pauses’ location with regard to clauses, predicting silent pauses’ duration.

The model including Semantic Verbal fluency (SVF) revealed marginally significant results. More specifically, a three-way significant interaction was found marginally significant, explaining 24% of the model’s variance, between narrative task, silent pauses location with regard to clauses and performance in SVF. Performance in SVF had a different effect on silent pauses duration, as a function of silent pauses location with regard to clauses; a pattern mostly apparent in picture description and for silent pauses detected between clauses (see Figure 4). A marginally significant negative main effect of performance in SVF on silent pauses duration was also found significant, explaining 23% of the model’s variance. Also, narrative task, silent pauses’ location and years of formal schooling revealed a negative effect on silent pauses’ duration, while total duration of narration has a significant positive effect (see Table 4 for patients’ performance in neuropsychological assessment and Table 5 for model’s summary). No other model revealed any significant interactions or main effects of neuropsychological tasks on silent pauses duration. For silent pauses’ frequency, mixed effects models did not yield any significant results.

**Table 4:** Descriptive data of neuropsychological assessment in group of patients with aphasia.

	Mean	(SD)	Range	(Min-Max)	
Boston Naming Test	11.22	8.7	13.00	0.00	13.00
Peabody Picture Vocabulary Test	19.78	8.9	31.00	9.00	31.00
Phonemic Fluency	9.36	8.7	29.00	0.00	29.00
Semantic Fluency	19.82	19.0	62.00	0.00	62.00
Complex Instructions	3.88	4.1	13.00	0.00	13.00

**Table 5:** Linear mixed effects model for performance in semantic verbal fluency, narrative task and silent pauses’ location predicting silent pauses’ duration.

Fixed effects					
	Est/Beta	SE	95% CI	t	*p*
Intercept	0.52	0.12	0.31–0.73	**4.501**	<0.001
Narrative task	−0.41	0.09	−0.59–−0.24	**−4.544**	<0.001
Silent pauses’ location	−0.55	0.10	−0.74–−0.36	**−5.720**	<0.001
*SVF*	−0.23	0.12	−0.44–−0.17	***−1.945***	0.06115
Total duration	0.18	0.06	0.08–0.29	**3.210**	0.00173
Age	−0.11	0.11	−0.30–0.08	−0.995	0.33216
Sex	−0.16	0.20	−0.52–0.21	−0.788	0.44068
Years of education	−0.21	0.09	−0.37–−0.05	**−2.338**	0.03074
Narrative Task x Silent pauses’ location	0.21	0.13	−0.04–0.48	1.576	0.11539
Narrative Task x SVF	0.13	0.09	−0.04–0.29	1.467	0.14270
Silent pauses’ location x SVF	0.15	0.10	−0.04–0.34	1.563	0.11841
*Narrative Task x Silent pauses’ location x SVF*	−0.24	0.13	−0.51–0.02	***−1.774***	0.07639
Random effects					
		Variance	S.D.		
Participant (Intercept)		0.10	0.32		
Model fit					
R^2^		Marginal	Conditional		
		0.14	0.24		

### 3.3 Third research question

**Figure 5:**
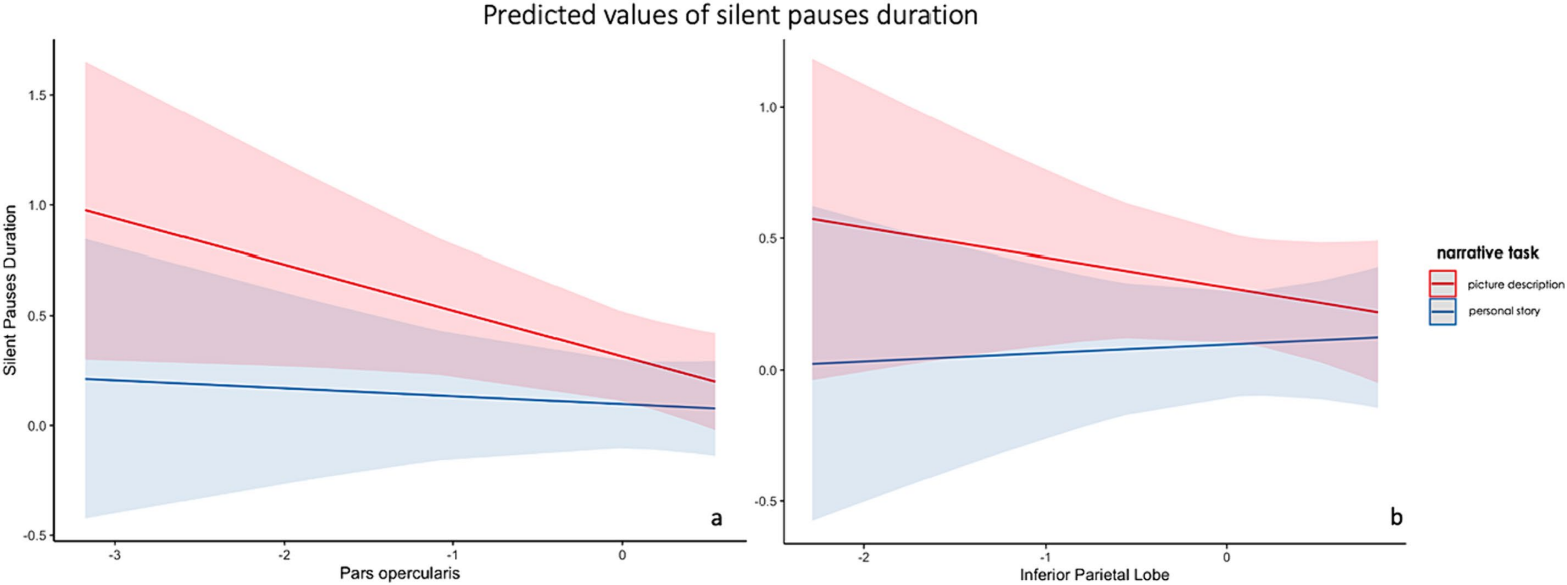
Effects plot for two-way interaction among narrative task and pars opercularis spared tissue **(A)** and inferior parietal lobe **(B)**, predicting silent pauses’ duration.

The first model including spared tissue of regions of interest of the dorsal stream for language revealed a significant positive two-way interaction between pars opercularis and narrative task, explaining almost 18% of the variance and a marginally significant positive two-way interaction between inferior parietal lobe and narrative task, explaining almost 14% of the model’s variance. In both interactions, spared tissue in aforementioned ROIs had a negative effect on silent pauses duration, a pattern apparent in picture description (see Figure 5). Spared tissue of Pars opercularis along with narrative task, age and sex revealed a negative effect on silent pauses’ duration (see model summary in Table 6).

**Table 6:** Linear mixed effects model for performance in Dorsal stream ROIs’ spared tissue and narrative task predicting silent pauses’ duration.

Fixed effects					
	Est/Beta	SE	95% CI	t	*p*
Intercept	0.32	0.10	0.13–0.50	**3.023**	0.005233
Inferior Parietal Lobe	−0.11	0.12	−0.33–0.11	−0.914	0.368096
Pars Opercularis	−0.21	0.10	−0.39–−0.03	**−2.081**	0.043996
Superior Temporal Gyrus	0.16	0.11	−0.04–0.40	1.480	0.148932
Narrative Task	−0.22	0.06	−0.34–−0.10	**−3.556**	0.000396
Age	0.15	0.07	0.02–0.27	**2.089**	0.045089
Sex	−0.54	0.17	−0.84–−0.23	**−3.086**	0.005082
Total Lesion Volume	−0.01	0.14	−0.26–0.25	−0.059	0.953473
*Inferior Parietal Lobe x Narrative Task*	0.15	0.08	−0.01–0.29	***1.914***	0.055983
Pars Opercularis x Narrative Task	0.17	0.07	0.03–0.31	**2.418**	0.015810
Superior Temporal Gyrus x Narrative Task	−0.10	0.07	−0.25–0.04	−1.375	0.169315
Random effects					
		Variance	S.D.		
Participant (Intercept)		0.12	0.35		
Model fit					
R^2^		Marginal	Conditional		
		0.10	0.22		

**Figure 6:**
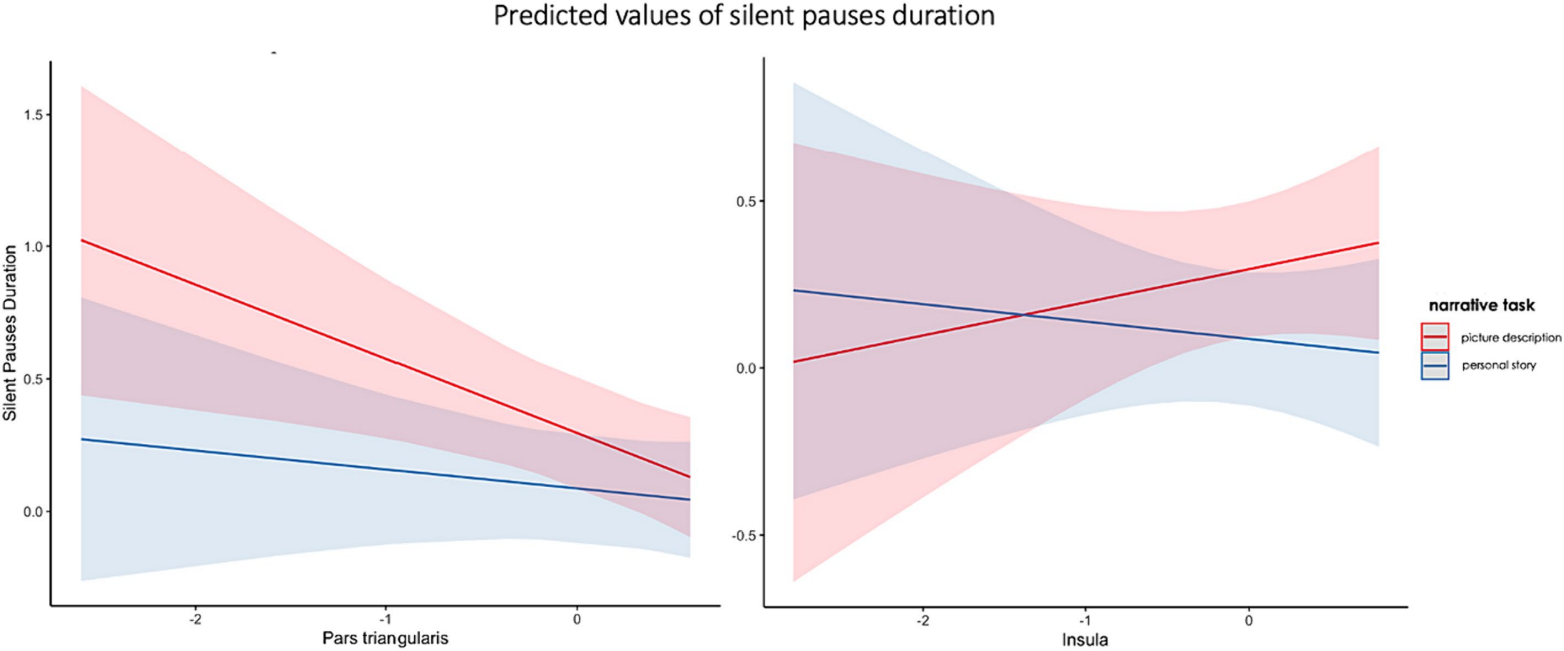
Effects plot for two-way interaction among narrative task and pars triangularis spared tissue **(A)** and insula **(B)**, predicting silent pauses’ duration.

The second model including spared tissue of regions of interest of the ventral stream for language revealed a significant positive two-way interaction between pars triangularis and narrative task, explaining almost 21% of the variance and a marginally significant positive two-way interaction between insula and narrative task, explaining almost 15% of the model’s variance. In the first interaction, spared tissue of pars triangularis had a negative effect on silent pauses duration, a pattern apparent in picture description. Spared tissue of Insula has a different effect on silent pauses duration, as a function of narrative task (see Figure 6). Pars Triangularis along with narrative task and sex revealed a negative effect on silent pauses’ duration, while age had a positive effect on silent pauses’ duration (see model summary in Table 7).

**Table 7:** Linear mixed effects model for performance in Ventral stream ROIs’ spared tissue and narrative task predicting silent pauses’ duration.

Fixed effects					
	Est/Beta	SE	95% CI	t	*p*
Intercept	0.30	0.10	0.13–0.50	**2.864**	0.007814
Insula	0.10	0.12	−0.33–0.11	0.836	0.409094
Pars Triangularis	−0.28	0.10	−0.39 - -0.03	**−2.691**	0.010045
Middle Temporal Gyrus	0.05	0.13	−0.04–0.40	0.276	0.784413
Narrative Task	−0.21	0.06	−0.34 - -0.10	**−3.419**	0.000656
*Age*	0.14	0.07	0.02–0.27	***1.905***	0.066684
Sex	−0.55	0.17	−0.84 - -0.23	**−3.190**	0.003978
Total Lesion Volume	−0.10	0.12	−0.26–0.25	−0.858	0.398602
*Insula x Narrative Task*	0.15	0.08	−0.01–0.29	***−1.846***	0.065225
Pars Triangularis x Narrative Task	0.21	0.08	0.03–0.31	**2.557**	0.010724
Middle Temporal Gyrus x Narrative Task	−0.08	0.06	−0.25–0.04	−1.264	0.206609
Random effects					
		Variance	S.D.		
Participant (Intercept)		0.12	0.35		
Model fit					
R^2^		Marginal	Conditional		
		0.09	0.21		

With regard to the models involving pause frequency, no significant interactions were found. However, there was a significant main effect of the spared tissue of the superior temporal gyrus on pause frequency, suggesting a positive relationship between the two variables [t(28.1) = 3.604, *p* = 0.0012].

## 4 Discussion

### 4.1 Summary of findings

Our results indicated that patients with aphasia exhibited significant differences in pause measures (frequency and duration), in both narrative conditions, compared to healthy speakers. Moreover, significant differences were identified within the patient group, revealing that patients’ speech output can be differentiated in distinct narrative tasks. More specifically, patients with aphasia exhibited significantly increased number of silent pauses in comparison to healthy speakers in both narrative tasks. Patients’ pause duration was longer in both tasks, although the difference was significant only for picture description. Within-group comparisons showed that silent pause frequency and duration were significantly increased in the picture description task for the patients, while healthy speakers revealed the opposite trend, i.e., increased number of silent pauses of longer duration in personal story.

With regard to our second research question, we found a marginally significant interaction between performance in semantic verbal fluency and narrative task, as a function of silent pauses’ location (i.e., between or within clauses), predicting silent pauses’ duration in the patients’ group. No significant results appeared for silent pauses’ frequency.

Finally, investigating our third research question, we found that silent pauses’ duration can be predicted by distinct ROIs spared tissue in the left hemisphere, as a function of narrative task. In particular, distinct interaction patterns between pars opercularis, pars triangularis, insula and inferior parietal with narrative task were revealed, predicting silent pauses’ duration. Analyses involving pause frequency did not reveal any significant interaction; however, there was an association between frequency of silent pauses and the superior temporal gyrus.

### 4.2 Silent pauses’ distinct patterns in the two narrative tasks

Our patients had increased silent pauses’ frequency and duration, compared to healthy speakers, in both tasks. This finding is far from surprising, based on previous evidence suggesting that patients with aphasia reveal disfluencies expressed by several metrics, such as silent pauses’ characteristics [see for example: (3, 12, 14)], but also speech rate [see for example: (55)]. It should be noted that our control participants were younger compared to our patients. Based on previously analyzed unpublished data (Angelopoulou, 2021), age does not significantly affect pause frequency and duration. Therefore, we can positively attribute the differences observed between our groups to the presence of aphasia, rather than age, even if the groups were not matched. Interestingly, silent pauses have been proposed as a sensitive marker capable of distinguishing between patients with moderate and severe aphasia (14), as well as between patients with latent aphasia and healthy speakers (12).

In our study, we employed two of the most common elicitation techniques, widely implemented in language research, each distinctly different from the other, a single picture description and a personal story narration of an illness/accident event. Our findings suggested that patients with aphasia may exhibit more disfluencies, reflected in silent pauses’ measures, in picture description compared to stroke story, while healthy speakers demonstrated the exact opposite pattern, that is increased silent pauses’ indices in personal story. Interpreting silent pauses as reflections of internal cognitive processing during speech output preparation, the current results contribute additional evidence to the notion that different speech genres involve distinct cognitive tasks with varying linguistic demands, grounded in diverse cognitive processes (16–18, 56).

Several researchers have previously stressed the importance of investigating different speech genres in aphasiological research [see (16, 57) for two reviews on discourse analysis in aphasia]. Picture description is considered to be of low cognitive challenge for healthy speakers, as illustrations tend to enhance discourse structure, however it seems that this is not the case for patients with aphasia. Naming difficulties are considered to be very common among patients with aphasia, even as residual deficits demonstrated by well-recovered, fully communicative stroke survivors (58). The appearance of specific visual stimuli that patients need to name may render the picture description a more demanding task [see also (4) for patients with PPA], as it is related with aspects of cognition, such as word finding processes and access to lexical/semantic representations along with organization of visual content and less on selective retrieval of information from semantic and episodic memory [see (59)].

Free narration, on the other hand, is related with several interactive aspects of cognition [for a review see (60)], as it is a fulfilled description of actions and events that have evolved over time (61). The formulation and production of a coherent and well-organized story based on personal memories has been generally characterized as a challenging process for healthy speakers (60, 62), as speakers seem to follow a decision-making process, accessing episodic and semantic memory (63), in order to seek and selectively retrieve specific groups of information and then organize them in chronological order, achieving temporal sequencing. Thus, cognitive aspects of decision-making, selective retrieval, planning, as well as manipulation and epoptic process, as the two major components within working memory (64, 65), are involved (66, 67). Nevertheless, in such narration the speakers are flexible to formulate their narration content without being restricted by external visual stimuli, as in the case of picture description. While we did not account for sentence semantic content and complexity in our study, it is plausible to assume that patients may formulate simpler stories with fewer details compared to healthy speakers. Consequently, this might contribute to a tendency for patients to exhibit fewer pauses.

### 4.3 Different association patterns between silent pauses and performance in neuropsychological tasks

To the best of our knowledge, no prior evidence exists for post-stroke aphasia about the connection between silent pauses’ measures derived from connected speech tasks and performance in neuropsychological tasks. The available evidence predominantly originates from studies involving patients with neurodegenerative diseases. Mack et al. (20) found a significant positive relation between overall pause rate in a fairy tale narration and naming ability in a combined verb and noun naming task in patients with PPA. The authors attributed their findings to potential differences in cognitive processes underlying noun and verb production in both naming and narration tasks. Similarly, Pistono et al. (5, 21) proposed a compensatory function for silent pauses in individuals with Mild Cognitive Impairment (MCI), as they also found a positive correlation between the rate of silent pauses, both between and within utterances, and performance in an anterograde autobiographical episodic memory task (5). In a subsequent study examining two narrative tasks (picture description and autobiographical narration), Pistono et al. (21) identified a positive correlation between semantic fluency and silent pauses derived from the picture description task. Additionally, the frequency of silent pauses in a memory-based narration was positively related to performance in verbal anterograde memory and visual recognition memory tasks. However, our analyses did not yield significant results. We found a marginally significant interaction between verbal fluency, silent pauses’ location and narrative task predicting silent pauses’ duration, as well as a marginally significant negative relation between verbal fluency and silent pauses’ duration.

It is noteworthy that in all the aforementioned studies the frequency of silent pauses appears to exhibit a positive correlation with performance in neuropsychological assessments, contrary to the expected negative correlation. It has been suggested that silent pauses reflect the effort exerted by speakers with cognitive deficits in accessing and retrieving information rather than indicating inherent difficulties, in other words they serve as a compensatory cognitive mechanism. In contrast, similar correlations in healthy participants demonstrate the opposite pattern, where the frequency of silent pauses displays a negative correlation with performance in various neuropsychological tests. Although we acknowledge that our results revealed marginally significant relations, the trend we found refers to silent pauses’ duration and not frequency. This observation may support the hypothesis that two distinct pause characteristics—frequency and duration—may offer different kinds of information, as discussed by Potagas et al. (4). This trend may strengthen the hypothesis that the occurrence of silent pauses could serve diverse roles in distinct narrative tasks and within different morphosyntactic locations in speech flow. In any case, our findings do not show a statistically significant association, but rather—as stated above—a trend. In this sense, this issue remains to be clarified and additional research is imperative to further elucidate the function of pauses, particularly concerning their location in different elicitation tasks.

### 4.4 Silent pauses correlation patterns with lesion indices

Our analyses showed significant interactions with spared tissue of left pars opercularis and inferior parietal lobe, indicating an inverse association pattern with silent pauses duration, mostly apparent in picture description and not stroke story. On the other hand, pars triangularis presented a negative association with silent pauses’ duration more apparent in picture description, while for insula there is a positive relation with picture description and a negative relation with stroke story. It should be noted that such interactions were not found for pause frequency, which was just positively associated with the amount of spared tissue of the superior temporal gyrus. This finding, i.e., a significant main effect in absence of interactions, is in contrast with the data derived from pause duration analyses, a fact that further supports the idea that these two metrics convey different types of information about the temporal organization of speech and should be treated as indices reflecting distinct aspects of narrative speech [see (4)].

Our findings suggested that silent pauses’ duration in picture description is related with both lesions in dorsal and ventral stream, while silent pauses in stroke story are mostly related with lesions in the ventral stream. If we hypothesize that silent pauses during picture naming reflect mostly word-finding difficulties but also speech planning process, they could be collimated with cognitive functions similar to a naming task [for a discussion see (68)]. Cookie theft picture is relatively simple and does not contain complex sequences of events. Thus, the relation with dorsal but also ventral stream areas could be attributed to the effort to successfully find and utter the right target words based on specific visual stimuli. By contrast, the duration of silent pauses in the stroke story is negatively related with remaining tissue in mostly left ventral stream, such as insula and pars triangularis. Stroke story is considered as a task of increased cognitive demands, requiring the selective retrieval of information from autobiographical memory and the temporal organization of specific events in a chronological order. It is assumed that this increased difficulty is reflected by longer silent pauses.

Lesions in ventral stream areas have been previously related with stroke story narration. Efthymiopoulou et al. (19) reported that speech rate in the stroke story was related to brain lesions affecting the temporofrontal extreme capsule fasciculus. According to the seminal paper by Saur et al. (45) on the dorsal and ventral pathways for language, as well as more recent evidence from the same team (69), the temporofrontal extreme capsule fasciculus (EmC) is a main component of the ventral stream, and connects temporal areas, mostly the middle temporal gyrus, with the pars triangularis (BA 45) of the inferior frontal gyrus, traversing through the extreme capsule and the insula. Advanced neuroimaging analysis of *in vivo* data have highlighted the crucial involvement of the left temporofrontal extreme capsule fasciculus in sound-to-meaning mapping and language comprehension (45). It is also assumed to facilitate the “*integration of information across different domains and modalities*” (69). Lesions in patients with acquired language disorders have indicated the significance of the left ventral path regions in the selective retrieval of verbal information from temporal cortices [(43); for an extended discussion see (19)]. Furthermore, evidence of the ventral stream involvement in information retrieval from the autobiographical memory comes from studies in amnestic patients (70, 71). According to the “multiple trace theory” (72–74) the left medial temporal lobe is assumed to be crucial for recovery of episodic memories.

Moreover, sparse evidence from studies in neurodegenerative diseases suggested that pauses may be related with phonological and articulatory processing, as they have related with atrophy in left hemisphere regions of dorsal stream, such as the pars opercularis, the precentral gyrus, the supramarginal gyrus, and the inferior parietal cortex (20). While it has been also suggested that they could reflect access autobiographical memory and organize discourse, as they have been related with atrophy in area BA10 of the left prefrontal cortex (5). Increased atrophy in prefrontal areas may serve as an indication of reduced attention capacity, necessary for the accomplishment of such an autobiographical narrative task. Thus, the appearance of increased frequency of silent pauses and their relation to increased atrophy may enhance the compensatory role of silent pauses in a demanding cognitive effort of organizing and successfully uttering the retrieved events. Finally, pause frequency in only the picture description task have also been related with atrophy in left anterior temporal lobe, which has been linked with a process of “an active lexical search” to semantic representations (21).

## 5 Conclusion

To conclude, our results further support the notion that distinct speech genres may be related with different underlying cognitive processes, as shown by the differentiation of pause association patterns depending on the elicitation task. In addition, we observed a differential effect of specific lesion loci on pause duration, as a function of narrative type; we therefore argue that the disturbance of the temporal aspects of speech (i.e., pauses) may have a specific neurological substrate involving left perisylvian regions. These findings further strengthen the idea that pause variables are meaningful indices and could be incorporated as useful metrics in the study -and eventually clinical practice- of acquired language impairment. Finally, our findings suggest a trend of an association between pause duration and verbal fluency; this remains to be confirmed by future studies. To the best of our knowledge, this is the first study presenting results on silent pause variables in two separate narrative tasks and their relationship with brain lesion loci in post-stroke aphasia.

## 6 Limitations and suggestions for further research

The present study introduces novel data on silent pauses in post-stroke aphasia; however, it is essential to acknowledge some limitations. The relatively modest sample size of 32 patients, with limited diversity in language deficits, may restrict the generalizability of the findings in the broaden population of patients with post-stroke aphasia. Expanding the cohort to include participants with a more diverse range of language deficits could offer a more comprehensive understanding of silent pause characteristics in post-stroke aphasia. Additionally, the focus on chronic post-stroke aphasia in the current study might overlook the dynamic changes in silent pauses during the acute and subacute phases. Examining patients at different stages of aphasia recovery could provide a more nuanced perspective on the temporal organization of speech in post-stroke aphasia. Moreover, implementing longitudinal designs could provide a more conclusive understanding of the association between silent pauses and cognitive performance throughout different phases of aphasia recovery. Another issue that needs further investigation is that of the association between pauses and cognitive functions, depending on speech genre and pause location. As shown above, our analyses did not yield significant results concerning this particular research question. Following the rationale of null hypothesis significant testing, such null results contradict previous findings derived from studies with different clinical populations. However, we argue that the observed trend, although not supported by statistical significance, merits further investigation with larger samples, in order for this issue to be clarified, at least for the case of post-stroke aphasia. Lastly, while in the present study we examined two distinct narrative tasks, exploring a broader range of speech genres, such procedural speech or well-known stories, could enhance the ecological validity of the findings. Despite these limitations, the study provides significant knowledge for understanding silent pauses in post-stroke aphasia and underscores the need for future research to address these constraints for a more comprehensive and nuanced investigation.

## Data availability statement

The datasets presented in this article are not readily available due to ethical restrictions, further enquiries can be directed to the corresponding author.

## Ethics statement

The studies involving humans were approved by Eginition Hospital, 1st Neurological Clinic, National and Kapodistrian University of Athens, Greece. The studies were conducted in accordance with the local legislation and institutional requirements. Written informed consent for participation in this study was provided by the participants’ legal guardians/next of kin.

## Author contributions

GA: Conceptualization, Data curation, Formal analysis, Investigation, Methodology, Visualization, Writing – original draft. DK: Conceptualization, Data curation, Formal analysis, Investigation, Methodology, Visualization, Writing – original draft. MV: Formal analysis, Investigation, Methodology, Writing – original draft. DT: Data curation, Validation, Writing – review & editing. GP: Data curation, Writing – review & editing. GV: Data curation, Methodology, Visualization, Writing – review & editing. EM: Formal analysis, Methodology, Resources, Writing – review & editing. EK: Data curation, Methodology, Writing – review & editing. VP: Writing – review & editing. NL: Formal analysis, Methodology, Visualization, Writing – review & editing. NK: Resources, Supervision, Writing – review & editing. AT: Data curation, Investigation, Writing – review & editing. SV: Data curation, Investigation, Writing – review & editing. DG: Conceptualization, Data curation, Methodology, Resources, Supervision, Writing – review & editing. SK: Formal analysis, Methodology, Resources, Writing – review & editing. CW: Investigation, Methodology, Writing – review & editing. MR: Investigation, Methodology, Supervision, Writing – review & editing. CP: Conceptualization, Data curation, Funding acquisition, Investigation, Methodology, Project administration, Supervision, Writing – review & editing.

## Footnotes

^3^www.mccauslandcenter.sc.edu/crnl/mricron/

^4^https://www.mccauslandcenter.sc.edu/mricrogl/

^5^http://www.r-project.org
